# Supplementary material for: Transcriptomic decoding of surface-based imaging phenotypes and its application to pharmacotranscriptomics
Source: Nat Commun. 2025 Jul 22;16:6727. doi: 10.1038/s41467-025-61927-3 (PMC12279933; doi:10.1038/s41467-025-61927-3)
Supplement: Supplementary file 1 — Supplementary Information [file 41467_2025_61927_MOESM1_ESM.pdf]

Supplementary Information

Supplementary Data Figure 1

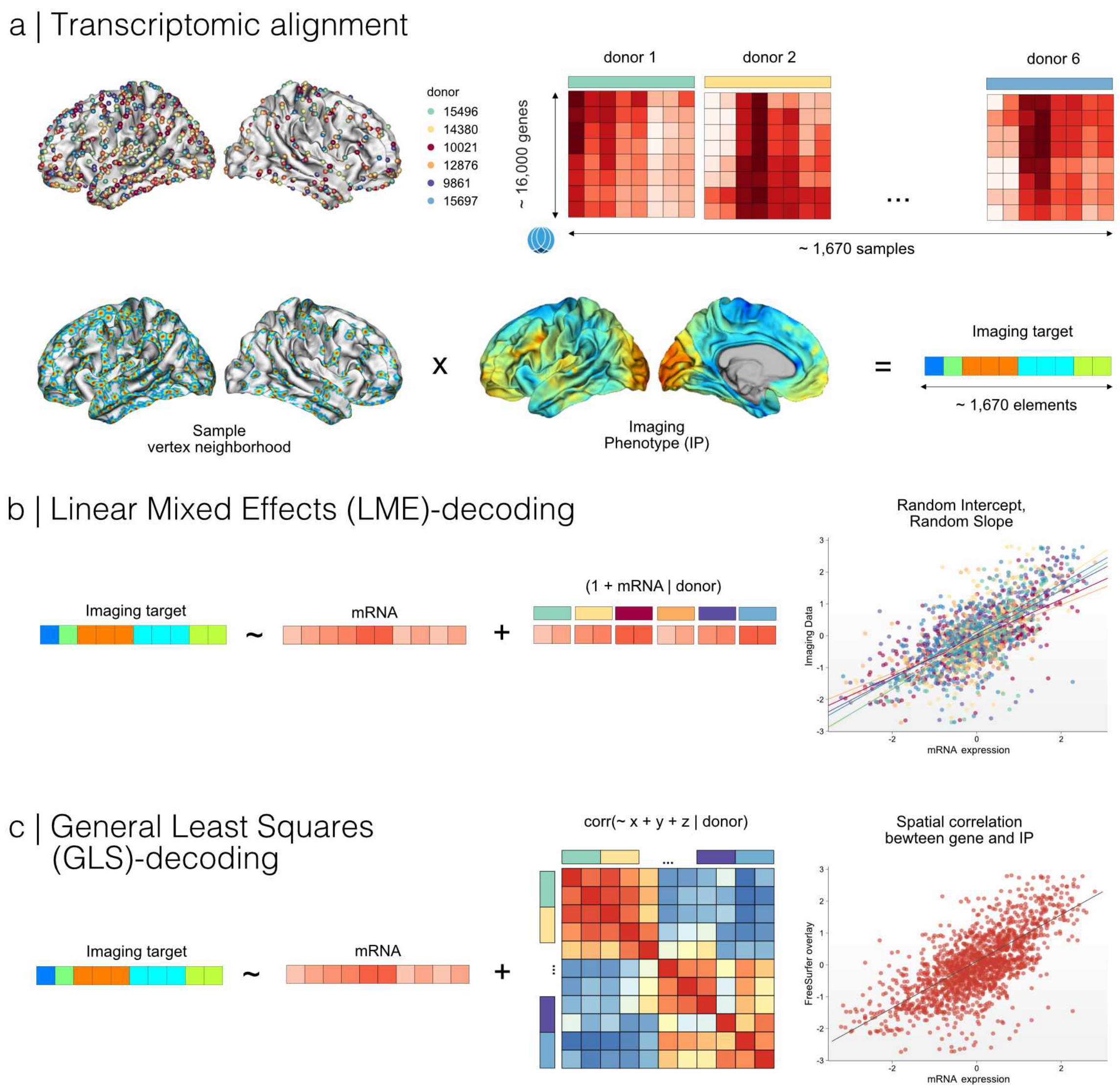

Supplementary Data Fig. 1. Gene expression decoding using Linear Mixed Effects (LME) and General Least Squares (GLS) analysis

a| Transcriptomic alignment of AHBA gene expression data and imaging-derived phenotype (IDP). At each of 1,670 AHBA sample vertices across the cortex, the values in the target IP were averaged within a geodesic vertex neighbourhood of 5mm, resulting in a spatial vector with 1,670 elements for the IDP. **b**| Linear Mixed Effects (LME)-decoding approach, in which the spatial correlation between a gene's expression profile and the IP was assessed via an LME model with random intercept and slope grouped by donor. **c**| General Least Squares (GLS)-decoding approach accounting for spatial autocorrelations, where the target map was predicted by the mRNA signature of each gene, covarying for a Gaussian autoregressive spatial correlation structure defined by the vertex' x, y, z coordinates, and donor as grouping factor.

Supplementary Data Figure 2

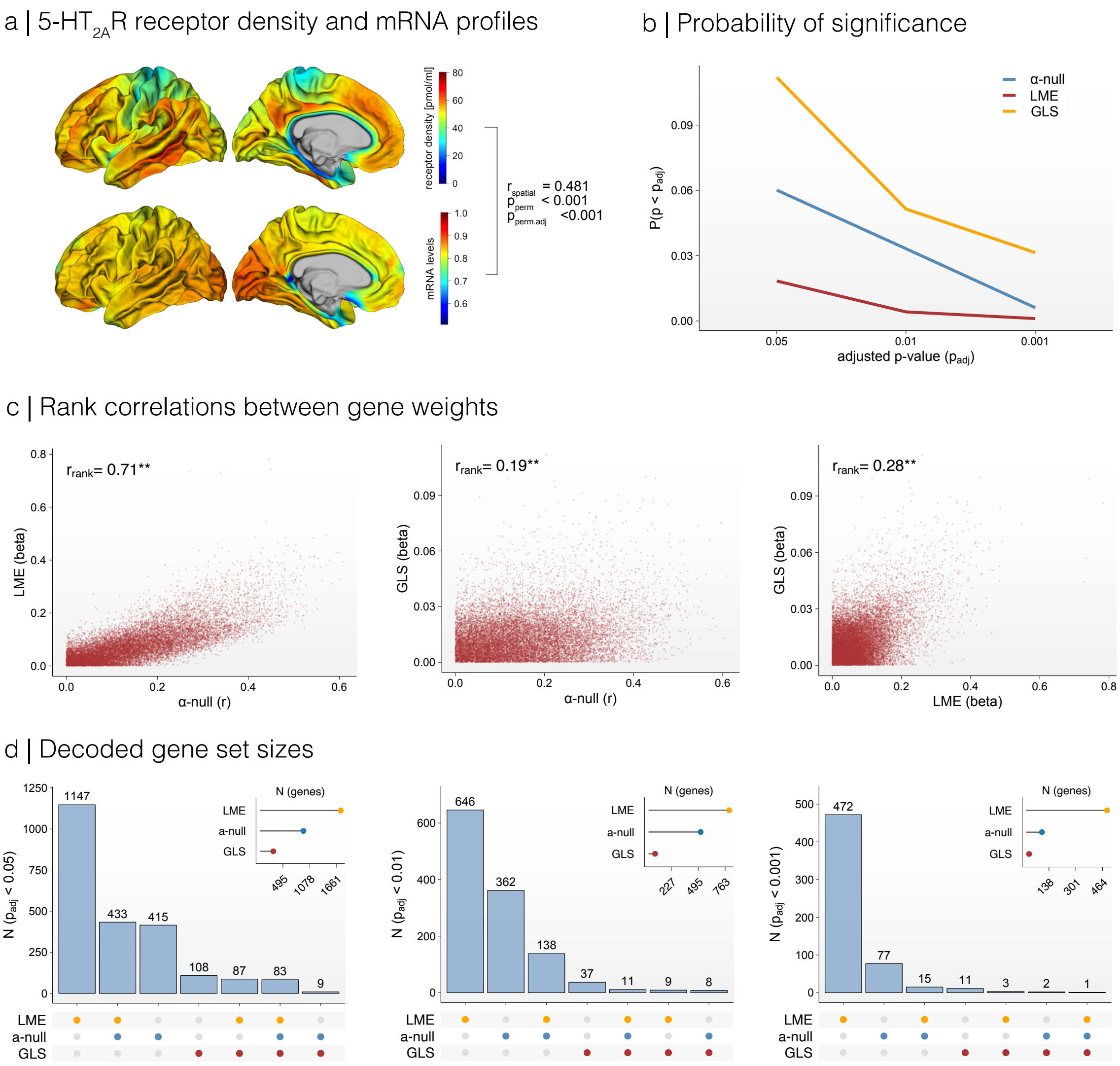

Supplementary Data Fig. 2. Surface-based transcriptomic decoding of the serotonergic 2A receptor (5-HT$_{2A}$R)

a| Spatial correlations between the average receptor density (B_{max}) of the 5-HT$_{2A}$R based on the high-resolution in vivo Positron Emission Tomography (PET) atlas of the Human serotonergic system provided by [15] (upper panel) and the predicted mRNA expression profile of the HTR2A gene (lower panel). **b|** False positive rate (FPR) of the **α**-null (blue), Linear Mixed Effects (LME, red), and General Least Squares (GLS, yellow) decoding techniques across different adjusted p-value thresholds (p_{adj}). For LME- and GLS-decoding, *p*-values were adjusted for multiple comparisons using False Discovery Rate (FDR) adjustments. For **α**-null decoding, adjusted permutation p-values ($p_{perm,adj}$) were used. **c|** Spearman rank correlations between the gene weights across approaches. Gene weights were determined by the slope of the regression line (i.e., beta) for the LME- and GLS-decoding approach, and by the spatial Pearson correlation between receptor density and gene expression maps for the **α**-null decoding approach. **d|** Intersection of gene sets with a significant transcriptomic association with the target pattern across various decoding methods. Gene set intersections and total set sizes are shown at an adjusted p-value threshold of 0.05 (left panel), 0.01 (middle panel), and 0.001 (right panel).

Supplementary Data Figure 3

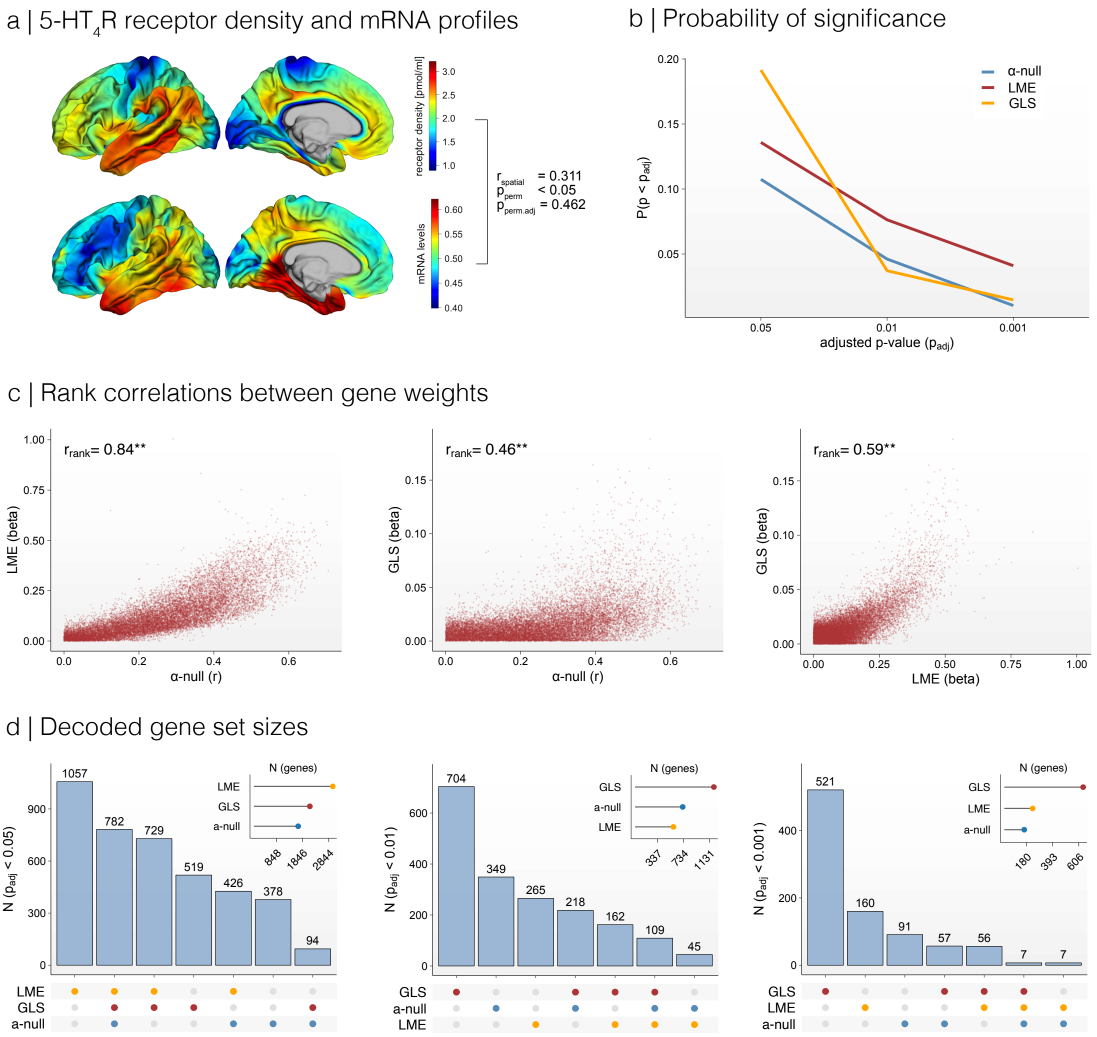

Supplementary Data Fig. 3. Surface-based transcriptomic decoding of the serotonergic receptor (5-HT4R)

a| Spatial correlations between the average receptor density (B_{max}) of the 5-HT4R based on the high-resolution in vivo Positron Emission Tomography (PET) atlas of the Human serotonergic system provided by [15] (upper panel) and the predicted mRNA expression profile of the HTR2A gene (lower panel). **b|** False positive rate (FPR) of the **α**-null (blue), Linear Mixed Effects (LME, red), and General Least Squares (GLS, yellow) decoding techniques across different adjusted p-value thresholds (p_{adj}). For LME- and GLS-decoding, *p*-values were adjusted for multiple comparisons using False Discovery Rate (FDR) adjustments. For **α**-null decoding, adjusted permutation p-values ($p_{perm,adj}$) were used. **c|** Spearman rank correlations between the gene weights across approaches. Gene weights were determined by the slope of the regression line (i.e., beta) for the LME- and GLS-decoding approach, and by the spatial Pearson correlation between receptor density and gene expression maps for the **α**-null decoding approach. **d|** Intersection of gene sets with a significant transcriptomic association with the target pattern across various decoding methods. Gene set intersections and total set sizes are shown at an adjusted p-value threshold of 0.05 (left panel), 0.01 (middle panel), and 0.001 (right panel).

Supplementary Data Figure 4

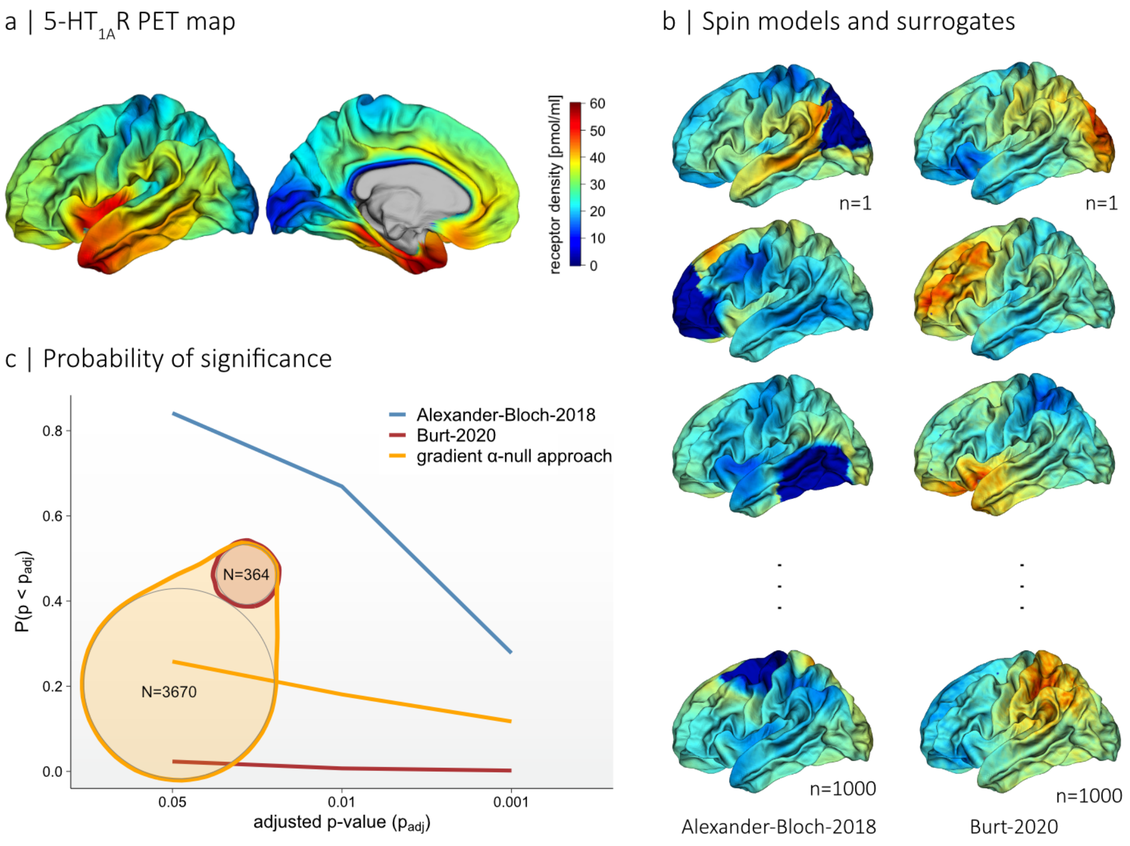

Supplementary Data Fig. 4. Comparison of the gradient-based **α**-null decoding approach with N=1,000 permutations of the target pattern

a| Positron Emission Tomography (PET) atlas of the Human serotonergic system provided by [15] for the 5-HT$_{1A}$ receptor (5-HT$_{1A}$R). **b**| Example spatial null models of the 5-HT$_{1A}$R target PET map using the spatial permutation null or spin modeling approach proposed by [21] (left panel), and the variogram matching approach proposed by [18] with optimization of the k_{nn} parameter (right panel). **c**| False positive rate (FPR) of the gradient-based **α**-null decoding approach (yellow), the spin modelling approach by Alexander-Bloch et al. (2018) (blue), and the variogram matching approach by Burt et al. (2020) (red). For all approaches, adjusted permutation p-values (p_{adj}) were used. Circles indicate the number of overlapping genes identified as being significantly associated with the target patterns at p_{adj}<0.05 by the gradient **α**-null decoding approach compared to the Burt et al. (2020) permutations of the target pattern.

Supplementary Data Figure 5

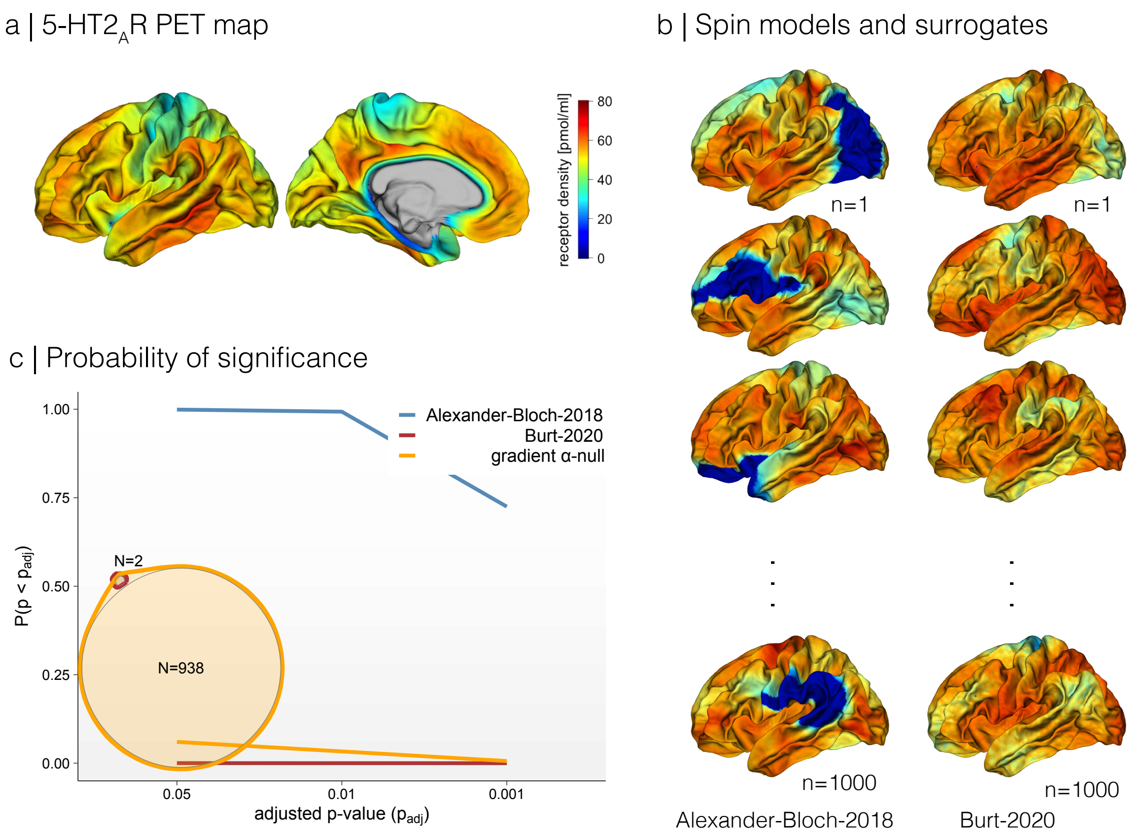

Supplementary Data Fig. 5. Comparison of the gradient-based **α**-null decoding approach with N=1,000 permutations of the target pattern

a| Positron Emission Tomography (PET) atlas of the Human serotonergic system provided by [15] for the 5-HT$_{2A}$ receptor (5-HT$_{2A}$R). **b**| Example spatial null models of the 5-HT$_{2A}$R target PET map using the spatial permutation null or spin modeling approach proposed by [21] (left panel), and the variogram matching approach proposed by [18] with optimization of the k_{nn} parameter (right panel). **c**| False positive rate (FPR) of the gradient-based **α**-null decoding approach (yellow), the spin modelling approach by Alexander-Bloch et al. (2018) (blue), and the variogram matching approach by Burt et al. (2020) (red). For all approaches, adjusted permutation p-values (p_{adj}) were used. Circles indicate the number of overlapping genes identified as being significantly associated with the target patterns at p_{adj}<0.05 by the gradient **α**-null decoding approach compared to the Burt et al. (2020) permutations of the target pattern.

Supplementary Data Figure 6

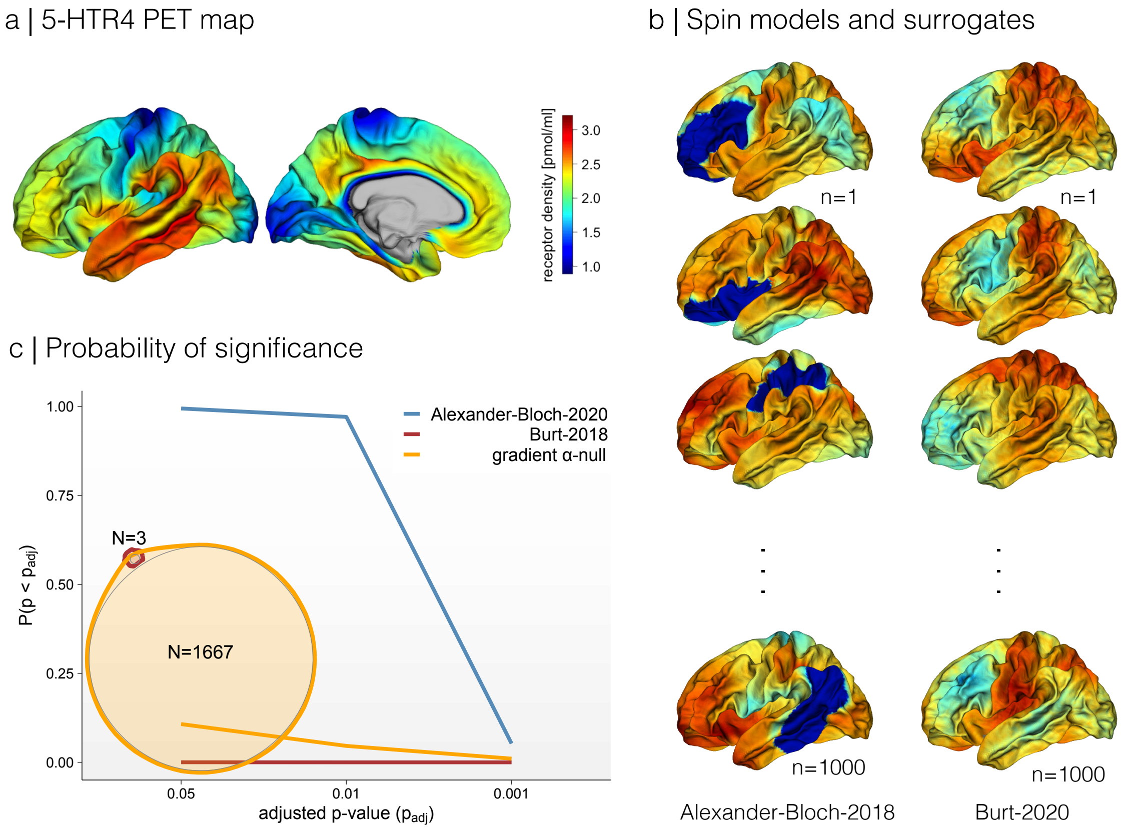

Supplementary Data Fig. 6. Comparison of the gradient-based **α**-null decoding approach with N=1,000 permutations of the target pattern

a| Positron Emission Tomography (PET) atlas of the Human serotonergic system provided by [15] for the 5-HT$_4$ receptor (5-HT$_4$R). **b**| Example spatial null models of the 5-HT$_4$R target PET map using the spatial permutation null or spin modeling approach proposed by [21] (left panel), and the variogram matching approach proposed by [18] with optimization of the k_{nn} parameter (right panel). **c**| False positive rate (FPR) of the gradient-based **α**-null decoding approach (yellow), the spin modelling approach by Alexander-Bloch et al. (2018) (blue), and the variogram matching approach by Burt et al. (2020) (red). For all approaches, adjusted permutation p-values (p_{adj}) were used. Circles indicate the number of overlapping genes identified as being significantly associated with the target patterns at p_{adj}<0.05 by the gradient **α**-null decoding approach compared to the Burt et al. (2020) permutations of the target pattern.

Supplementary Data Figure 7

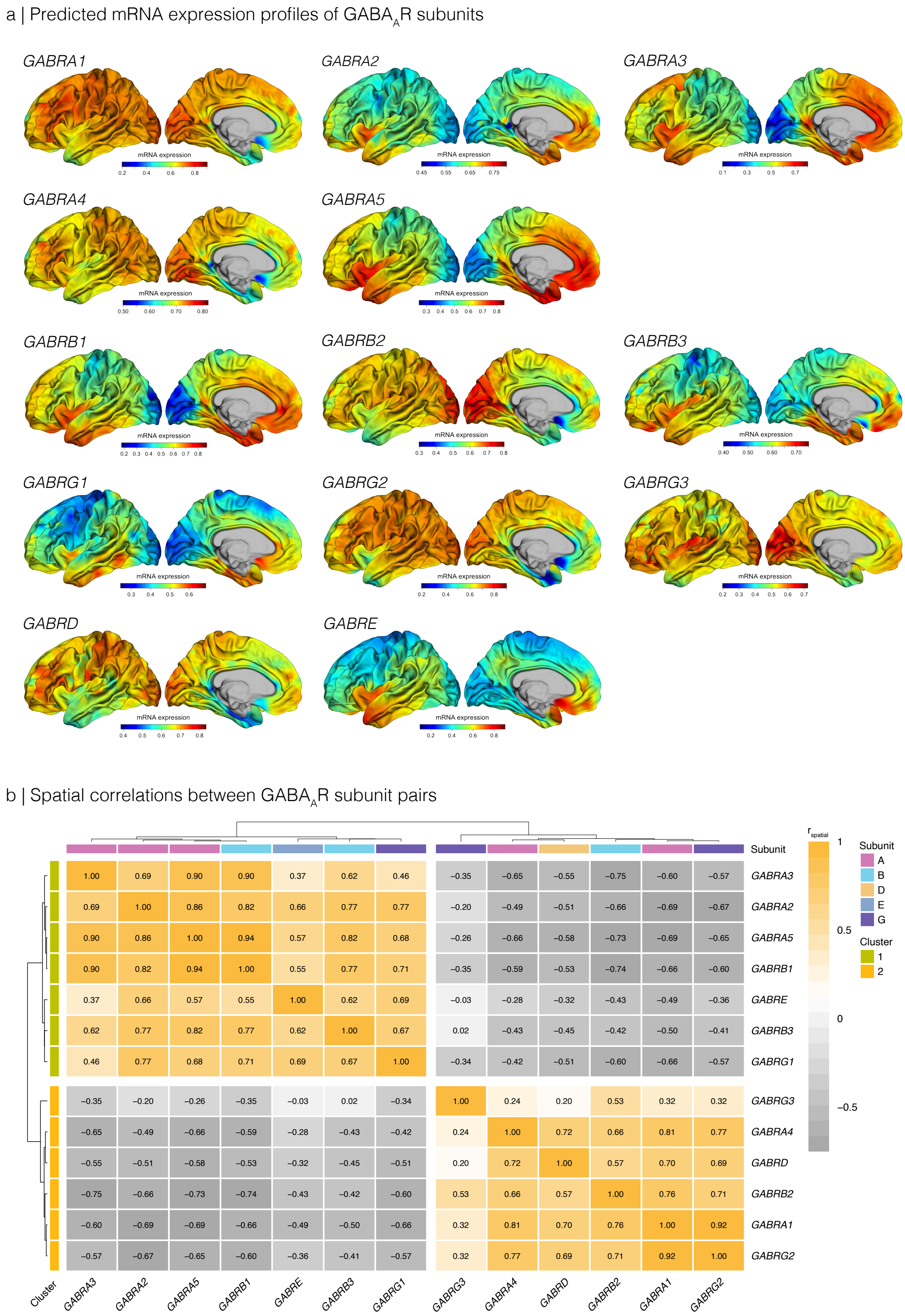

Supplementary Data Fig. 7. Predicted cortical mRNA expression signatures of GABA$_A$ receptor subunit genes

a| Predicted cortical mRNA expression signatures of 13 GABA$_A$ receptor (GABA$_A$R) subunit genes. **b**| Hierarchical clustering of GABA$_A$R subunit genes based on their cortical mRNA expression profiles. Values indicate the spatial correlation between subunit pairs. Subunit genes were allocated to two clusters with a similar transcriptomic landscape.

Supplementary Data Figure 8

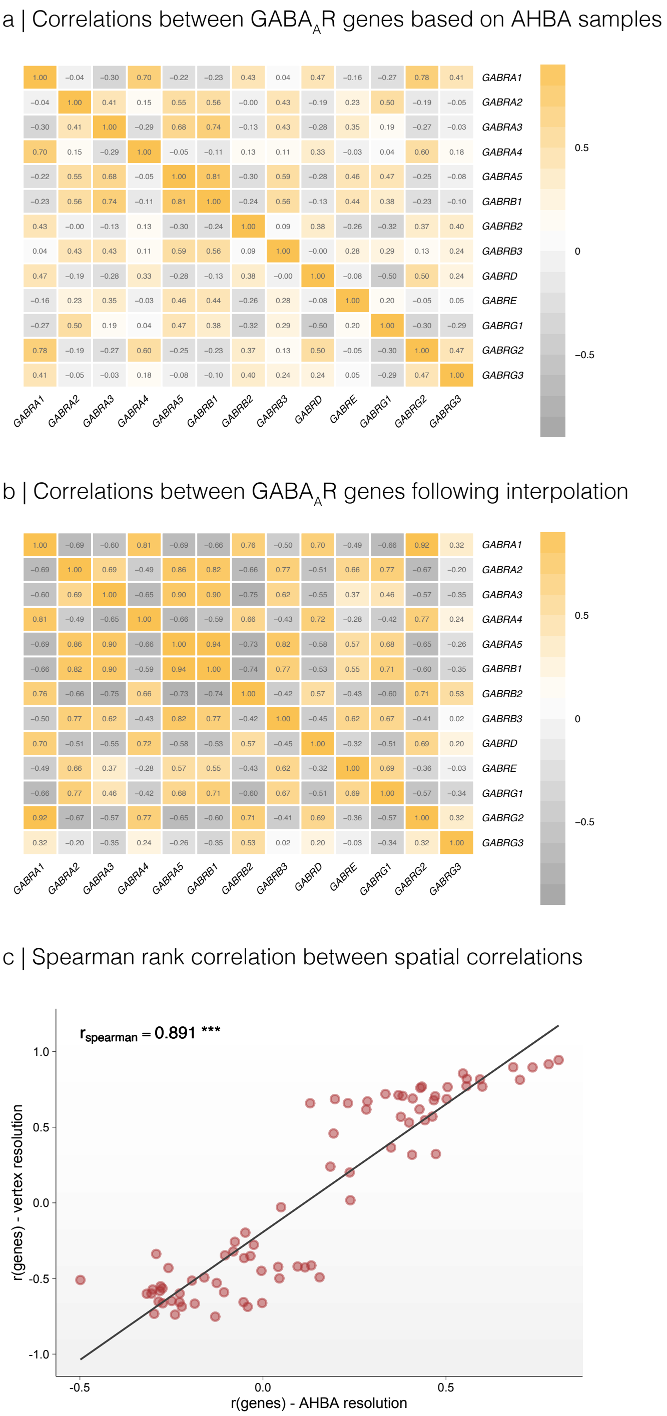

Supplementary Data Fig. 8. Differential stability of genes pre and post spatial interpolation

a| Spatial correlation matrix of Pearson correlation coefficients between the gene expression signatures of the 13 GABA$_A$ receptor subunit genes prior to spatial interpolation, i.e., in native resolution of the AHBA atlas. **b**| Spatial correlation matrix of Pearson correlation coefficients between gene expression signatures following spatial interpolation in FreeSurfer fsaverage6 (41k) resolution. **c**| Spearman rank correlation across spatial correlations between gene pairs in AHBA and vertex resolution. *** indicates a p-value < 0.01.

Supplementary Data Figure 9

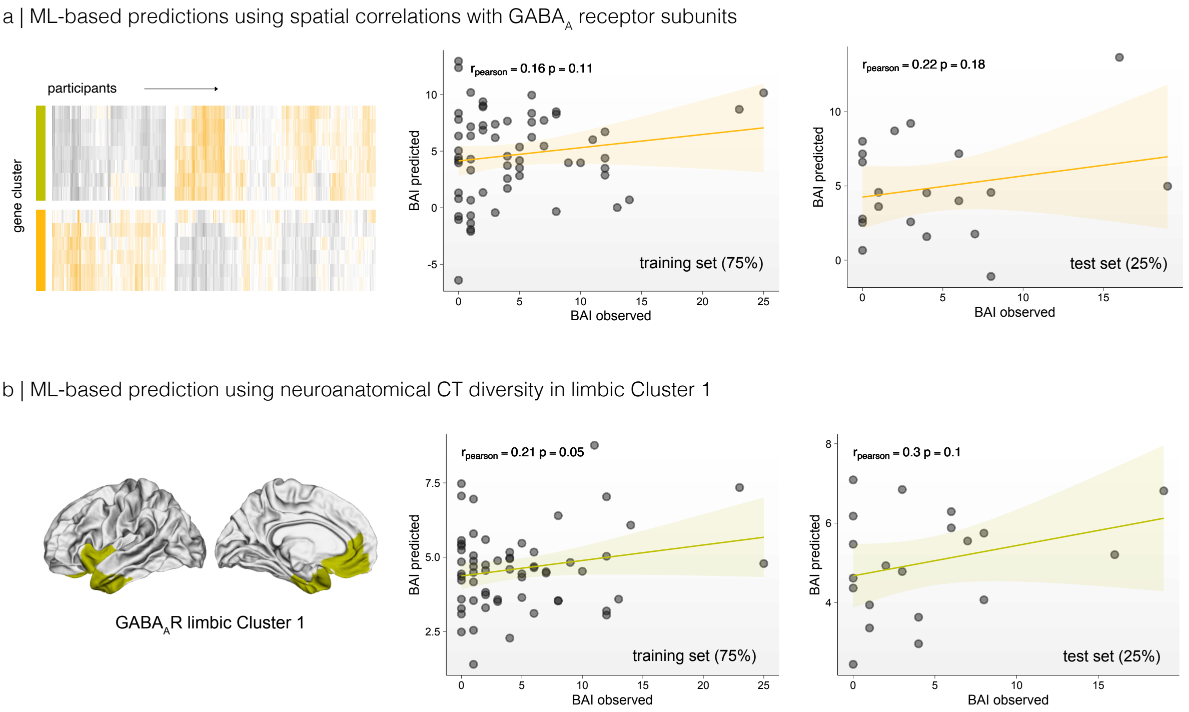

Supplementary Data Fig. 9. Machine Learning (ML)-based prediction of anxiety levels in adults based on the transcriptomic alignment between imaging phenotypes and GAB$_A$-R subunit genes using a linear model.

a| ML-predictions of self-reported Beck Anxiety Inventory (BAI) scores based on the matrix of spatial correlations between IDPs and the spatially interpolated cortical mRNA expression signatures of the 13 GAB$_A$R subunit genes (left panel). Correlations between observed and predicted BAI scores resulting from the final models in the training set (75%, middle panel) and test set (25%, right panel). The shaded area indicates the 95% confidence interval around the regression line. **b**|. ML-predictions of BAI scores based on the individuals' total degree of neuroanatomical diversity in CT within brain regions with high mRNA expression levels of GABA$_A$R subunit Cluster 1 (i.e., the limbic cluster) in the training set (75%, final model, middle panel) and test set (25%, right panel). Masks were generated by applying a threshold to the mean expression patterns of genes within each cluster, anchored at the 80th percentile of their distribution across the cortex (see Fig. 5c). The shaded area indicates the 95% confidence interval around the regression line.

Supplementary Data Figure 10

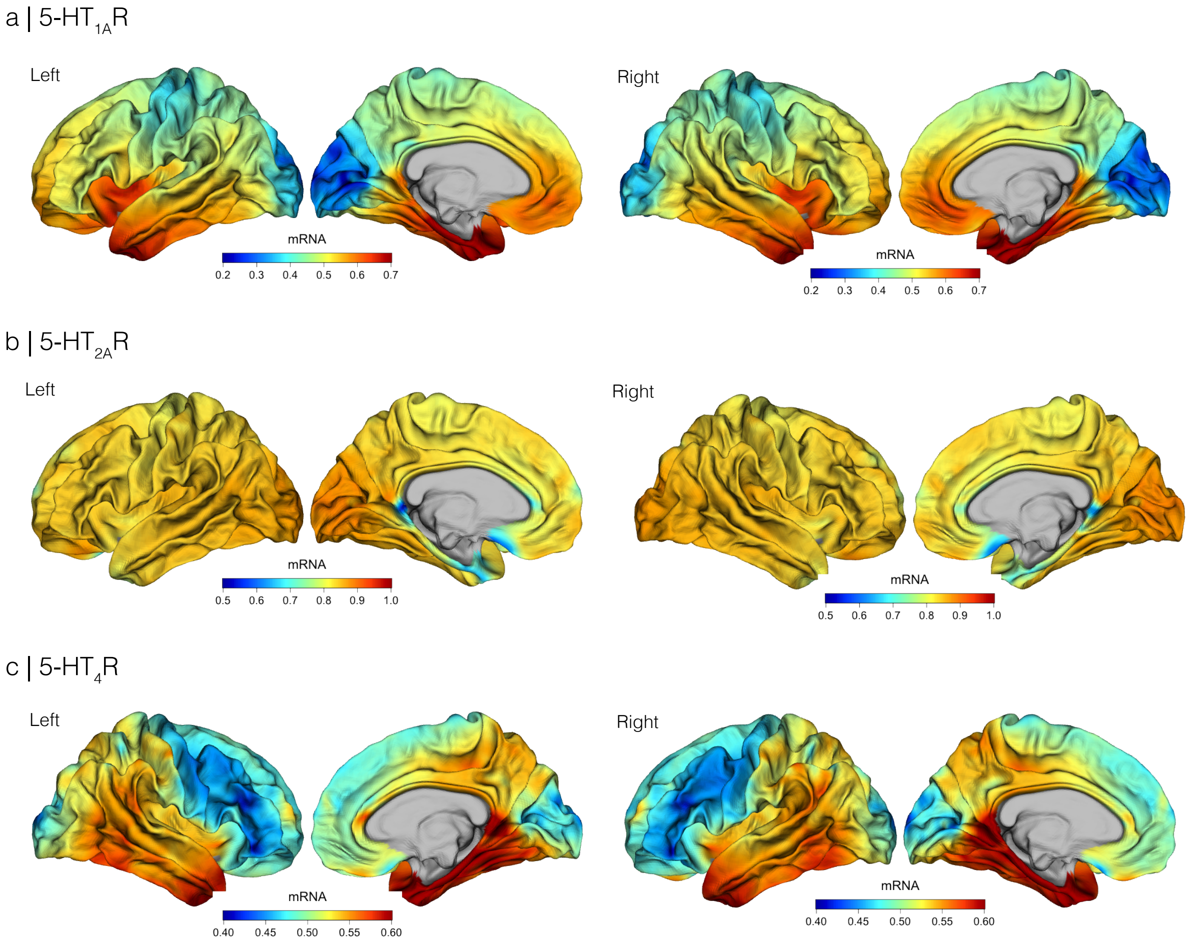

Supplementary Data Fig. 10. Spatially interpolated mRNA expression profiles for both hemispheres

a| Serotonergic 5-HT_{1A} receptor (5-HT_{1A}R), **b**| Serotonergic 5-HT_{2A} receptor (5-HT_{2A}R), **c**| Serotonergic 5-HT_4 receptor (5-HT_4R). Note. Left denotes the left hemisphere, Right denotes the right hemisphere.

Supplementary Data Figure 11

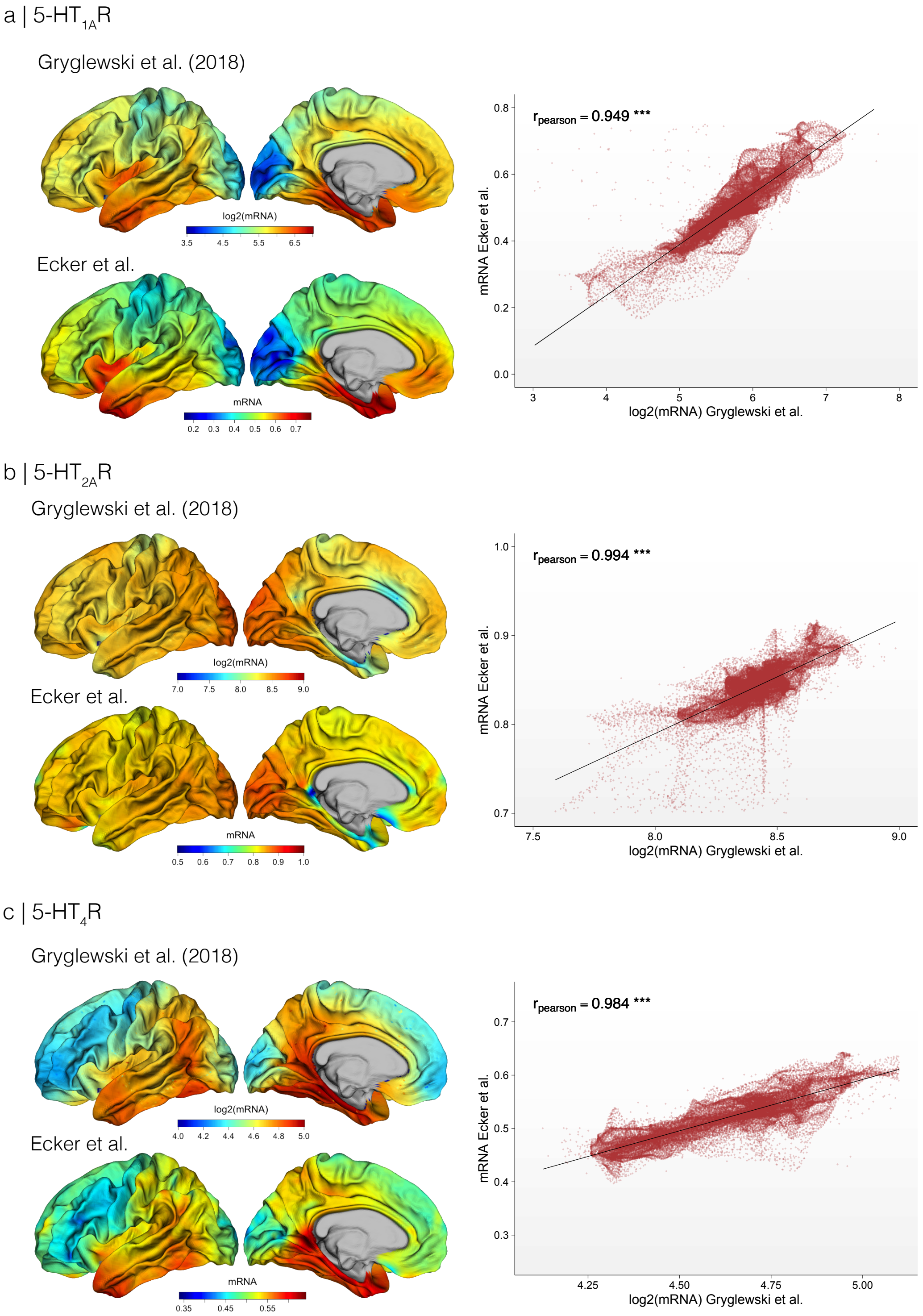

Supplementary Data Fig. 11. Comparison between the spatially interpolated mRNA expression profiles published by Gryglewski et al. (2018) [6] and the maps derived in the current study **a**| Serotonergic 5-HT1A receptor (5-HT1AR), **b**| Serotonergic 5-HT2A receptor (5-HT2AR), **c**| Serotonergic 5-HT4 receptor (5-HT4R). Note. Left panel shows the spatially interpolated mRNA expression patterns presented in the current study (lower panel) in comparison to [6] (upper panel). The scatterplots in the right panel show the bivariate Pearson correlations between the vertex-level mRNA expression signatures across publications. *** indicates a p-value < 0.01 (one-tailed).

Supplementary Data Figure 12

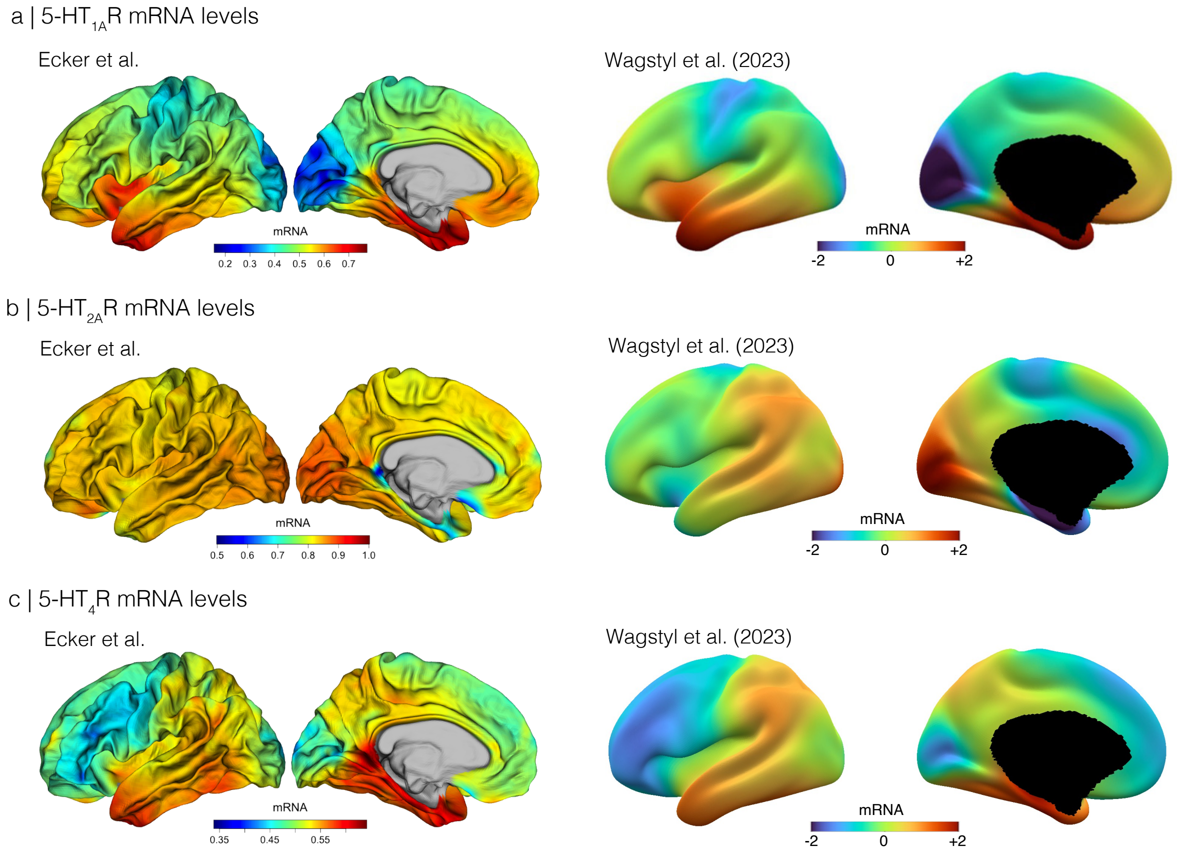

Supplementary Data Fig. 12. Comparison between the spatially interpolated mRNA expression profiles utilized in the current study compared to the maps released by Wagstyl et al. (2023) [20]

a| Serotonergic 5-HT_{1A} receptor (5-HT_{1A}R), **b**| Serotonergic 5-HT_{2A} receptor (5-HT_{2A}R), **c**| Serotonergic 5-HT_4 receptor (5-HT_4R). Note. Left panel shows the spatially interpolated mRNA expression patterns presented in the current study. Right panel shows the smoothed mRNA expression signatures released by Wagstyl et al. (2023) [20]

Supplementary Data Figure 13

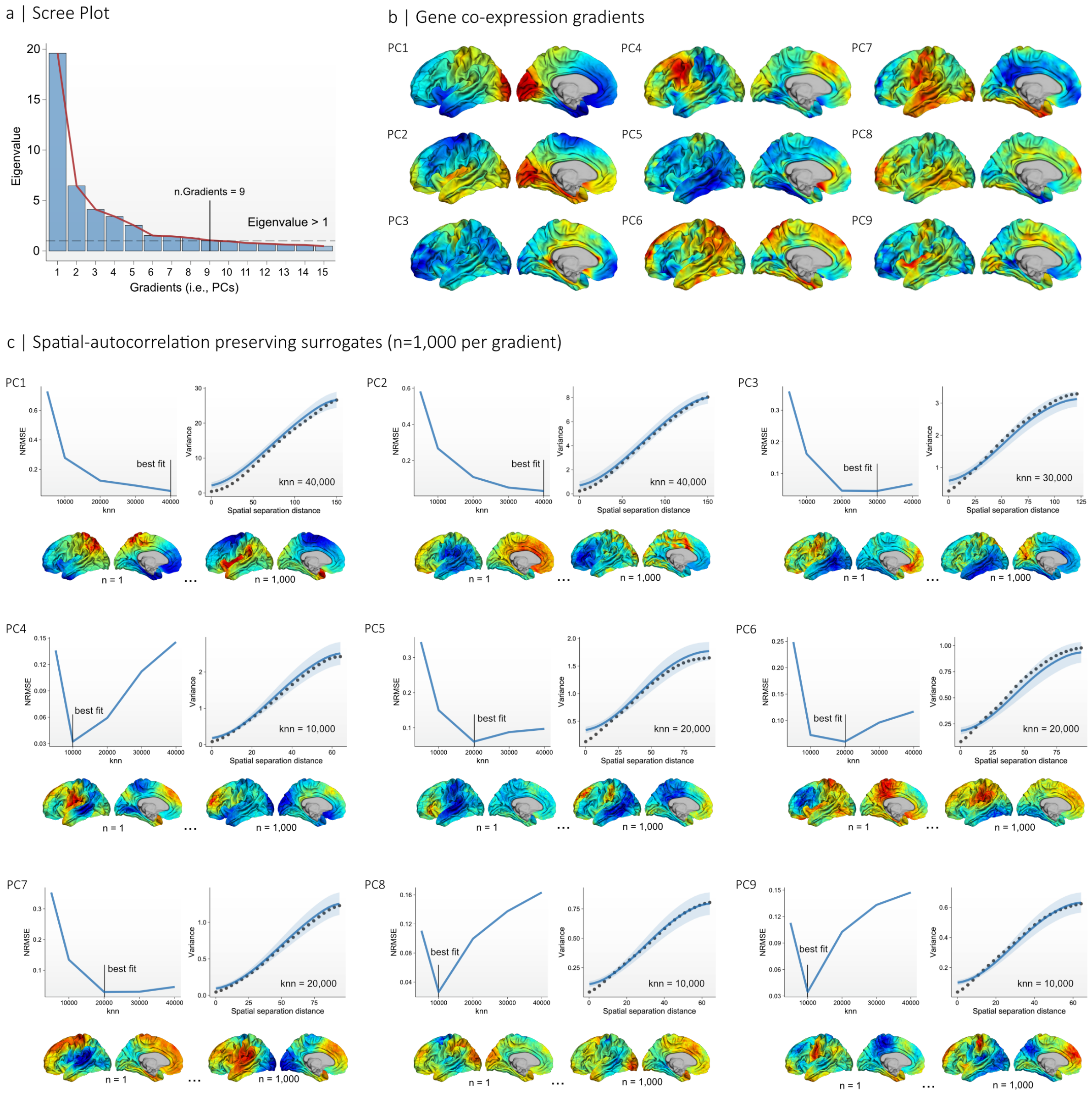

Supplementary Data Fig. 13. Gene co-expression gradients across the cortical surface
a| Scree plot resulting from the Singular Value Decomposition (SVD) of the gene-by-gene covariance matrix across the predicted vertex-level mRNA expression profiles. Nine co-expression gradients exhibited an Eigenvalue larger than one (dotted line), and together explained ~41% of variability in gene expression across the cortex. **b**| Cortical distribution of co-expression gradients of the nine components with an Eigenvalue larger than one. **c**| Spatial autocorrelation (α)-preserving null models (i.e., surrogates [18]) created for each gradient pattern under optimization of the K-nearest-neighbour (knn) parameter. The bar indicates the model with the best fit based on the model with the minimum normalized root squared error (NRMSE) between predicted and observed variance across separation distances. The left panels shown the NRMSE for different knn values. The right panel shows the model fit (i.e. Variogram) of the best-fitting model. For each gradient pattern, 1,000 surrogate maps were generated. The first and last permuted gradient patterns are shown in the lower panel. Data are presented as mean (blue line) ± standard deviation (shaded area) across N=100 surrogate fits.

Supplementary Data Figure 14

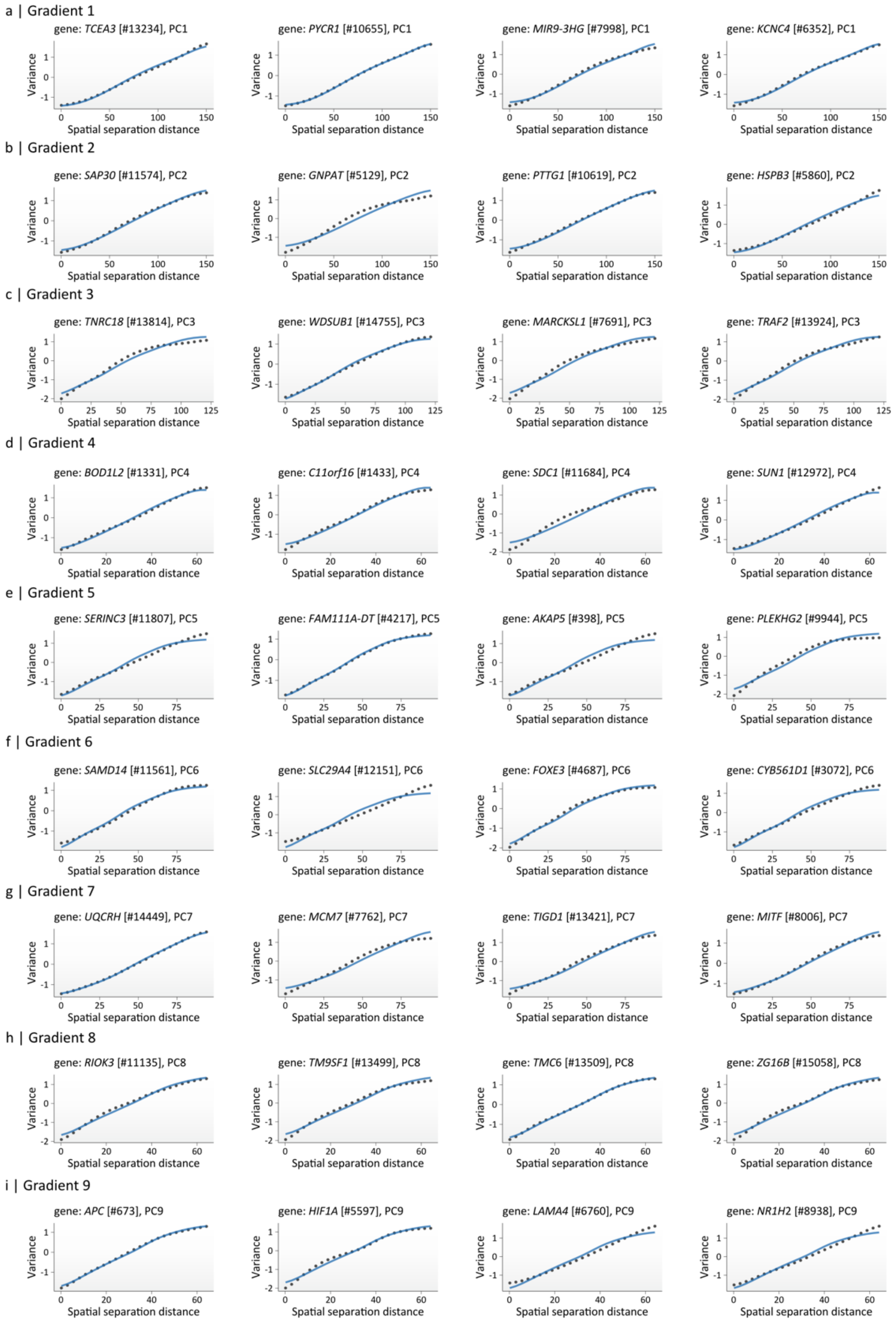

Supplementary Data Fig. 14. Empirical variograms of the nine co-expression gradients (blue line) and those of individual gene expression signatures (dotted black line) across a random selection of four genes per gradient pattern. Note. PC: principal component pattern, [#]: internal gene index.

Supplementary Data Tables

Supplementary Data Table 1. Medication status for the N=279 participants examined in the present study

**Unknown n (%)**	**No n (%)**	**Yes n (%)**	**Medication**
155 (56%)	103 (37%)	21 (8%)<br>One: n=21<br>Two: n=4<br>Three: n=1	▪ Antidepressant (n=4)<br>▪ 3 Selective serotonin reuptake inhibitor (SSRI)<br>▪ 1 Tetracyclic antidepressant (TeCA)<br>▪ Antiepileptics (n=2; no additional information)<br>▪ Anxiolytics (n=1; no additional information)<br>▪ Drugs used in addictive disorder (n=1; no additional information)<br>▪ Hypnotics and sedatives (n=3)<br>▪ 3 Melatonin<br>▪ Other analgesics and antipyretics (n=5)<br>▪ 1 Opioids<br>▪ 4 Other analgesic and antipyretics<br>▪ Psychostimulants and other drugs used to treat attention-deficit/hyperactivity disorder (ADHD) (n=10)<br>▪ 2 Atomoxetine<br>▪ 8 Methylphenidate hydrochloride

Note. n: number of participants.

Supplementary Data Table

Supplementary Data Table 2. Information on co-occurring mental health conditions in our cohort

**Co-occurring condition**	**Further information**
Attention-deficit/hyperactivity-disorder	Clinical Assessment of Attention Deficit-Adult (CAT) Scale (https://paa.com.au/product/cat-a/)<br>• Do not meet DSM-5 criteria for ADHD (n=196)<br>• Meet DSM-5 criteria for ADHD (n=25)<br>• Missing information (n=58)
Intellectual Disability	• Mild intellectual disability, with IQ between 50 and 74 (n=25)<br>• No intellectual disability (n=254)
Depression	DAWBA Depression Band (DSM IV & ICD-10); ordinal score 0-5<br>• 0: n=157<br>• 1: n=40<br>• 2: n=5<br>• 3: n=7<br>• 4: n=2<br>• 5: n=1<br>• missing: n=67
ADHD	DAWBA ADHD Band (DSM-IV); ordinal score 0-5<br>• 0: n=97<br>• 1: n=10<br>• 2: n=4<br>• 3: n=8<br>• 4: n=4<br>• 5: n=2<br>• missing: n=154
Anxiety	DAWBA Pooled Anxiety Disorder Band (DSM-IV); ordinal score 0-5<br>• 0: n=25<br>• 1: n=159<br>• 2: n=30<br>• 3: n=10<br>• 4: n=10<br>• 5: n=1<br>• missing: n=44
Behavioral Disorders	DAWBA Behavioral Disorder Band (DSM-IV & ICD-10), ordinal score 1-5<br>• 0: n=0<br>• 1: n=60<br>• 2: n=47<br>• 3: n=5<br>• 4: n=0<br>• 5: n=1<br>• missing: n=166

Note. DAWBA: Development and Well-Being Assessment [50]. To maximise the number of observations with available DAWBA scores, we utilized a built-in DAWBA algorithm recommended by the instrument's author, to create scores based on reports by either the primary caregiver, proband, or a combination thereof. Scores represent standard, computer-generated categories that describe the probability of a person meeting diagnostic criteria [50]: 0: $p < 0.1\%$, 1: $p \sim 0.5\%$, 2: $p \sim 3\%$, 3: $p = 15\%$, 4: $p = 50\%$, and 5: $p \geq 70\%$.

Supplementary Data Table

Supplementary Data Table 3. Summary scores for measures of anxiety and depression in our cohort

Rating Scale	n	mean	SD
BYI-II Anxiety self-rated	66	8.77	6.99
BAI self-rated	82	4.56	5.32
BYI-II Anxiety parent-rated	62	7.11	6.55
BAI parent-rated	9	5.89	6.45
BYI-II Depression self-rated	67	7.04	7.53
BDI self-rated	82	4.02	5.22
BYI-II Depression parent-rated	64	6.03	7.49
BDI parent-rated	9	15.67	12.79

Note. For children younger than 11 years, parents completed the depression and anxiety subscales of the Beck Youth Inventories (BYI-II [43]). Adolescents (aged 12-17 years) were given the depression and anxiety subscales of the BYI-II as self-report. In adults, self-reports of symptoms associated with depression and anxiety were measured using (respectively) the Beck Depression Inventory – Second Edition (BDI-II [44]), and the Beck Anxiety Inventory (BAI [45]). n: number of individuals, SD: standard deviation

EU-AIMS LEAP Consortium

Full list of all consortium members

EU-AIMS LEAP Group:

Jumana Ahmad[1], Sara Ambrosino[2], Bonnie Auyeung[3], Tobias Banaschewski[4], Simon Baron-Cohen[3], Sarah Baumeister[4], Christian F. Beckmann[5], Sven Bölte[6], Thomas Bourgeron[7], Carsten Bours[5], Michael Brammer[1], Daniel Brandeis[4], Claudia Brogna[8], Yvette de Bruijn[5], Jan K. Buitelaar[5], Bhismadev Chakrabarti[3], Tony Charman[9], Ineke Cornelissen[5], Daisy Crawley[1], Flavio Dell'Acqua[1], Guillaume Dumas[10], Sarah Durston[2], Christine Ecker[11], Jessica Faulkner[1], Vincent Frouin[12], Pilar Garces[13], David Goyard[12], Lindsay Ham[14], Hannah Hayward[1], Joerg Hipp[13], Rosemary Holt[3], Mark H. Johnson[15], Emily J.H. Jones[15], Prantik Kundu[16], Meng-Chuan Lai[17], Xavier Liogier D'Ardhuy[13], Michael V. Lombardo[18], Eva Loth[1], David J. Lythgoe[19], René Mandl[2], Andre Marquand[5], Luke Mason[1], Maarten Mennes[5], Andreas Meyer-Lindenberg[4], Caroline Moessnang[4], Nico Müller[4], Declan G.M. Murphy[1], Bethany Oakley[1], Laurence O'Dwyer[5], Marianne Oldehinkel[5], Bob Oranje[2], Gahan Pandina[20], Antonio M. Persico[21], Barbara Ruggeri[22], Amber Ruigrok[3], Jessica Sabet[1], Roberto Sacco[8], Antonia San José Cáceres[1], Emily Simonoff[23], Will Spooren[24], Julian Tillmann[25], Roberto Toro[7], Heike Tost[4], Jack Waldman[3], Steve C.R. Williams[19], Caroline Wooldridge[19], and Marcel P. Zwiers[5].

[1]Department of Forensic and Neurodevelopmental Sciences, Institute of Psychiatry, Psychology, and Neuroscience, King's College London; London, UK
[2]University Medical Center Utrecht, Utrecht University; Utrecht, Netherlands.
[3]Autism Research Centre, Department of Psychiatry, University of Cambridge; Cambridge, United Kingdom.
[4]Department of Child and Adolescent Psychiatry, Central Institute of Mental Health, Medical Faculty Mannheim, University of Heidelberg; Mannheim, Germany.
[5]Department of Cognitive Neuroscience, Donders Institute for Brain, Cognition and Behaviour, Radboud University Nijmegen Medical Centre; Nijmegen, Netherlands.
[6]Center for Neurodevelopmental Disorders, Karolinska Institutet; Stockholm, Sweden.
[7]Human Genetics and Cognitive Functions, Institut Pasteur, UMR3571 CNRS, Université Paris Cité, IUF; Paris, France
[8]Department of Paediatrics, The Catholic University of America, University Campus Bio-Medico; Rome, Italy.
[9]Clinical Child Psychology, Department of Psychology, Institute of Psychiatry, Psychology and Neuroscience, King's College London; London, United Kingdom.
[10]CHU Sainte-Justine Research Center, Department of Psychiatry, University of Montreal; Montreal, Canada.
[11]Department of Child and Adolescent Psychiatry, Psychosomatics and Psychotherapy, University Hospital Frankfurt, Goethe University; Frankfurt am Main, Germany.
[12]Neurospin Centre CEA; Gif sur Yvette, France.
[13]Roche Pharma Research and Early Development, Neuroscience, Ophthalmology and Rare Diseases, Roche Innovation Center Basel; Basel, Switzerland.
[14]Regulatory Affairs, Pharmaceutical Development, F. Hoffmann-La Roche Pharmaceuticals; Basel, Switzerland.
[15]Centre for Brain & Cognitive Development, University of London; London, United Kingdom.
[16]Department of Radiology, Icahn School of Medicine at Mount Sinai; New York, New York, USA.
[17]Child and Youth Mental Health Collaborative, Centre for Addiction and Mental Health and The Hospital for Sick Children, Department of Psychiatry, University of Toronto; Toronto, Canada.
[18]Laboratory for Autism and Neurodevelopmental Disorders, Center for Neuroscience and Cognitive Systems @UniTn, Istituto Italiano di Tecnologia; Rovereto, Italy.
[19]Department of Neuroimaging, Institute of Psychiatry, Psychology and Neuroscience, King's College London; London, United Kingdom.
[20]Janssen Research & Development; Titusville, New Jersey, USA.
[21]Child and Adolescent Neuropsychiatry, Department of Biomedical, Metabolic and Neural Sciences, University of Modena and Reggio Emilia; Modena, Italy.
[22]Social, Genetic and Developmental Psychiatry Centre, Institute of Psychiatry, Psychology and Neuroscience, King's College London; London, United Kingdom.

[23]Department of Child and Adolescent Psychiatry, Institute of Psychology, Psychiatry and Neuroscience, King's College London; London, United Kingdom.
[24]Roche Pharmaceutical Research and Early Development, NORD Discovery and Translational Area, Roche Innovation Center Basel; Basel, Switzerland.
[25]F. Hoffmann La Roche, Innovation Center Basel; Basel, Switzerland.

The primary contact for the EU-AIMS LEAP Group is Declan G. Murphy (Email: pa-dmurphy@kcl.ac.uk).
