# Supplementary material for: Transcriptomic decoding of surface-based imaging phenotypes and its application to pharmacotranscriptomics
Source: Nat Commun. 2025 Jul 22;16:6727. doi: 10.1038/s41467-025-61927-3 (PMC12279933; doi:10.1038/s41467-025-61927-3)
Supplement: Supplementary file 2 — Reporting Summary [file 41467_2025_61927_MOESM2_ESM.pdf]

nature portfolio

Corresponding author(s): Christine Ecker

Last updated by author(s): Jun 4, 2025

Reporting Summary

Nature Portfolio wishes to improve the reproducibility of the work that we publish. This form provides structure for consistency and transparency in reporting. For further information on Nature Portfolio policies, see our Editorial Policies and the Editorial Policy Checklist.

Statistics

For all statistical analyses, confirm that the following items are present in the figure legend, table legend, main text, or Methods section.

n/a	Confirmed	
☐	☒	The exact sample size (*n*) for each experimental group/condition, given as a discrete number and unit of measurement
☐	☒	A statement on whether measurements were taken from distinct samples or whether the same sample was measured repeatedly
☐	☒	The statistical test(s) used AND whether they are one- or two-sided<br>*Only common tests should be described solely by name; describe more complex techniques in the Methods section.*
☐	☒	A description of all covariates tested
☐	☒	A description of any assumptions or corrections, such as tests of normality and adjustment for multiple comparisons
☐	☒	A full description of the statistical parameters including central tendency (e.g. means) or other basic estimates (e.g. regression coefficient) AND variation (e.g. standard deviation) or associated estimates of uncertainty (e.g. confidence intervals)
☐	☒	For null hypothesis testing, the test statistic (e.g. *F*, *t*, *r*) with confidence intervals, effect sizes, degrees of freedom and *P* value noted<br>*Give P values as exact values whenever suitable.*
☒	☐	For Bayesian analysis, information on the choice of priors and Markov chain Monte Carlo settings
☒	☐	For hierarchical and complex designs, identification of the appropriate level for tests and full reporting of outcomes
☐	☒	Estimates of effect sizes (e.g. Cohen's *d*, Pearson's *r*), indicating how they were calculated

Our web collection on statistics for biologists contains articles on many of the points above.

Software and code

Policy information about availability of computer code

Data collection	Data was collected within the EU-AIMS Longitudinal European Autism Project (LEAP) and contained structural MRI and phenotypic assessments of healthy controls.
Data analysis	The project utilized open source code and libraries/packages written in R, python, and cpp. We leveraged existing, open source code (as specified in the methods); and also generated new code (see Code Availability statement: The software generated for this project is freely available on github (https://github.com/christineecker/fsdecode, and https://github.com/christineecker/fsnulls)).

For manuscripts utilizing custom algorithms or software that are central to the research but not yet described in published literature, software must be made available to editors and reviewers. We strongly encourage code deposition in a community repository (e.g. GitHub). See the Nature Portfolio guidelines for submitting code & software for further information.

Data

Policy information about availability of data

All manuscripts must include a data availability statement. This statement should provide the following information, where applicable:

- Accession codes, unique identifiers, or web links for publicly available datasets
- A description of any restrictions on data availability
- For clinical datasets or third party data, please ensure that the statement adheres to our policy

The raw neuroimaging data are protected and are not available due to data privacy laws. The pre-processed neuroimaging data are available here: https://gin.g-

node.org/sphache/DecodeGABAData. All scripts are provided as publicly available repositories: https://github.com/christineecker/fsnulls, https://github.com/christineecker/fsdecode, and https://gin.g-node.org/sphache/DecodeGABA/src/main.

Research involving human participants, their data, or biological material

Policy information about studies with human participants or human data. See also policy information about sex, gender (identity/presentation), and sexual orientation and race, ethnicity and racism.

Reporting on sex and gender	We have adhered to common standards with regards to referring and reporting on sex differences.
Reporting on race, ethnicity, or other socially relevant groupings	There are no references or concerns with regards to race, ethnicity, or other socially relevant groupings within the manuscript.
Population characteristics	The study examined healthy controls exclusively, some of which had a mild intellectual disability (i.e., IQ < 70) ranging in age from 7 to 31 years.
Recruitment	Recruitment occured within the EU-AIMS Longitudinal European Autism Project (LEAP), which is described in detail in PMID 28649313.
Ethics oversight	The study was approved by national and local ethics review boards at each site, and was carried out to Good Clinical Practice (ICH GCP) standards. More specifically, at each recruitment center, namely (i) King's College London & University of Cambridge, London-Central and Queen Square Health Research Authority, Research Ethics Committee (Ref. Nr. 13/LO/1156), (ii) Radboud University Medical Centre & University Medical Centre Utrecht, Institute Ensuring Quality and Safety Committee on Research Involving Human Subjects Arnhem-Nijmegen (Ref. Nr. 2019-5942), (iii) Medical University Mannheim, Medical Ethics Commission II (Ref. Nr. 2020-547N), and (iv) Bio-Medical University Campus Rome, Ethics Committee De Roma (Ref. Nr. 18/14 PAR ComET CBM).

Note that full information on the approval of the study protocol must also be provided in the manuscript.

Field-specific reporting

Please select the one below that is the best fit for your research. If you are not sure, read the appropriate sections before making your selection.

☐ Life sciences ☒ Behavioural & social sciences ☐ Ecological, evolutionary & environmental sciences

For a reference copy of the document with all sections, see nature.com/documents/nr-reporting-summary-flat.pdf

Behavioural & social sciences study design

All studies must disclose on these points even when the disclosure is negative.

Study description	The data was provided by the EU-AIMS Longitudinal European Autism Project (LEAP), which is described in detail in PMID 28649313. The study was originally designed to parse heterogeneity in autism spectrum disorders, and compare neurodevelopmental trajectories to typical brain development. The sample provides deep phenotypic and clinical assessments, in addition to MRI measures. The data used in this study are quantitative.
Research sample	The project utilized a total of N=279 typically developing controls recruited across 6 European sites that included (i) King's College London, (ii) University of Cambridge, (iii) Radboud University Medical Centre, (iv) University Medical Centre Utrecht, (v) Bio-Medical University Campus Rome, and (vi) Medical University Mannheim. The neuroimaging cohort comprised a total of N=279 control participants, split into 254 typically developing participants (90 female, 164 male) and 25 individuals with mild intellectual disability (ID; 11 female, 14 male; defined by a Full-Scale IQ (FSIQ) between 50 and 74) between the ages of 7 and 31 years (mean age = 17.32 + 5.91 years). Level of intellectual abilities was assessed using the Wechsler Abbreviated Scales of Intelligence—Second Edition, WASI-II or—in countries where the WASI is not translated (i.e. The Netherlands, Germany and Italy)—the four-subtest short forms of the German, Dutch or Italian WISC-III/IV for children or WAIS-III/IV for adults.
Sampling strategy	Participants were recruited from a variety of sources including existing volunteer databases, existing research cohorts, clinical referrals from local outpatient centres, special needs schools, mainstream schools and local communities. Note that the large-scale study from which our data were derived performed power calculations to determine the appropriate sample size to address that study's research questions, see PMID: 28649312).
Data collection	Data for the study was acquired on conventional 3T MRI scanners, and using standard questionnaires. More specifically, measures of anxiety and depression were obtained for children younger than 11 years, where parents completed the depression and anxiety subscales of the Beck Youth Inventories (BYI-II 31). Adolescents (aged 12-17 years) were given the depression and anxiety subscales of the BYI-II as self-report. In adults, self-reports of symptoms associated with depression and anxiety were measured using (respectively) the Beck Depression Inventory – Second Edition (BDI-II) and the Beck Anxiety Inventory (BAI).
Timing	January 2014 and March 2017
Data exclusions	Exclusion criteria included significant hearing or visual impairments not corrected by glasses or hearing aids, a history of alcohol and/

Data exclusions	or substance abuse or dependence in the past year and the presence of any MRI contraindications (e.g. metal implants, braces, claustrophobia) or failure to give informed written consent to MRI scanning (or to provide contact details for a primary care physician at centres where this is a pre-condition for scanning).
Non-participation	One participant withdrew consent after the study completion and has been removed from the dataset.
Randomization	n/a

Reporting for specific materials, systems and methods

We require information from authors about some types of materials, experimental systems and methods used in many studies. Here, indicate whether each material, system or method listed is relevant to your study. If you are not sure if a list item applies to your research, read the appropriate section before selecting a response.

Materials & experimental systems

n/a	Involved in the study	
☒	☐	Antibodies
☒	☐	Eukaryotic cell lines
☒	☐	Palaeontology and archaeology
☒	☐	Animals and other organisms
☒	☐	Clinical data
☒	☐	Dual use research of concern
☒	☐	Plants

Methods

n/a	Involved in the study	
☒	☐	ChIP-seq
☒	☐	Flow cytometry
☐	☒	MRI-based neuroimaging

Plants

Seed stocks	n/a
Novel plant genotypes	n/a
Authentication	n/a

Magnetic resonance imaging

Experimental design

Design type	n/a
Design specifications	n/a
Behavioral performance measures	n/a

Acquisition

Imaging type(s)	T1-weighted structural MRI images
Field strength	3T
Sequence & imaging parameters	High-resolution structural T1-weighted volumetric images were acquired with full head coverage, at 1.2 mm thickness with 1.2 x 1.2 mm in-plane resolution
Area of acquisition	whole-brain

Diffusion MRI ☐ Used ☒ Not used

Preprocessing

Preprocessing software	FreeSurfer version 6.0.0

Normalization	Standard normalization implemented in FreeSurfer software
Normalization template	FreeSurfer fsaverage6 surface template
Noise and artifact removal	n/a
Volume censoring	n/a

Statistical modeling & inference

Model type and settings	Clustering of cortical thickness maps based on their transcriptomic association with GABA-A receptor subunit genes.
Effect(s) tested	Spatial correlation between imaging-derived phenotype and cortical expression patterns of GABA-A receptor subunit genes.

Specify type of analysis: ☒ Whole brain ☐ ROI-based ☐ Both

Statistic type for inference (See Eklund et al. 2016)	Non-parametric assessment of spatial correlations within the spatial-null modelling framework.
Correction	maxT correction and FDR

Models & analysis

n/a	Involved in the study
☒	☐ Functional and/or effective connectivity
☒	☐ Graph analysis
☒	☐ Multivariate modeling or predictive analysis
